# American Proctologic Society

**Published:** 1909-09

**Authors:** Lewis H. Adler

**Affiliations:** Philadelphia


### SOCIETY PROCEEDINGS.

### American Proctologic Society

*Eleventh Annual Meeting, held at Atlantic City, N. J., June 7 and 8, 1909.*

Reported by LEWIS H. ADLER Jr.. M. D., Philadelphia, Secretary.

The president, Dr. George B. Evans, of Dayton, O., in the chair.

Officers elected for the ensuing year: president, Dwight H. Murray, Syracuse; vice-president, T. Chittenden Hill, Boston; secretary-treasurer, Lewis H. Adler, Jr., Philadelphia. Executive council: George B. Evans, Dayton, chairman; Dwight H. Murray, Syracuse; Louis J. Hirschman, Detroit; Lewis H. Adler. Jr., Philadelphia

The place of meeting for 1910 is St. Louis, Mo., Headquarters, Planters Hotel, June 6 and 7, 1910.

The following were elected fellows of the society: Charles S. Gilman, 419 Boylston Street, Boston, Mass.; Donley C. Hawley, Burlington, Vt., and Frank C. Yeomans, 19 E. 45th Street, New York City.

The following is an abstract of the principal papers read.

President's Address—Progress in Proctology, George B. Evans, A.M., M.D., Dayton, O.

Who stated that not many years since, the creation of proctology as a specialty was frowned upon; for an indefinite period what was known of and what was done for diseases of the rectum was largely empiric, and not due to special knowledge or scientific study.

A few of us at least, can remember when it was the rule among general practitioners to make no special effort to determine the pathology of diseases of the rectum; in fact, it was believed unbecoming the dignity of a high-classed, high-toned medical gentleman to so lightly esteem modesty as to ask for the privilege of seeking the naked truth. Without attempting to make a diagnosis, opium and lead wash, with catharsis, was deemed a sufficient treatment for any case. Little was taught in medical colleges of these diseases, for little was known and no special desire to learn much concerning them seemed to exist. But, fortunately, in the natural evolution of this specialty, this ignorance and indifference in the main, has been eliminated, and this field of work has assumed that of an accredited, and justifiable specialty. No longer do we have to contend with the non-recognition of serious pathology, because of interposed modesty, ignorance and criminal indifference. A knowledge of the importance of being able to diagnose and treat intelligently diseases of the rectum is now considered essential for every general practitioner, and all this as a result of the creation of proctology by men who have made special effort to develop this field of work. The credit is due to such men as Adler, Allingham, Ball, Gripps, Edwards, Earle, Gant, Martin, Pennington, Kelsey, Matthews and others. To them are we indebted for progressive proctology.

As a matter of course, our pathology of this area is of necessity a modern pathology, and our knowledge of valves, varicosities, neoplasms, ulcerations and suppurations, are not based on hypothetical ideas of a quarter of a century since, but instead on the rather exact revelations of laboratory findings. The import of the presence of staphylococci, gonococci, colon bacilli and tubercle bacilli, is equally as much importance to the rectal surgeon, as is the microscopical proof of the malignancy or benignity of a bit of tissue. With what greater assurance the proctologist approaches examinations of rectal diseases than did the physician of some years since. With a wide open field, if necessary, the aid of anesthesia, the protoscope and the laboratory, there is usually not much difficulty in making a diagnosis,—a diagnosis inseparably linked with its dependents,—treatment and prognosis. Under the influence of progressive proctologic work, ignorance and indifference to the recognition and treatment of rectal diseases is rapidly disappearing from the average medical man, as well as from the average layman. As a result of which the sum total of human suffering is immeasurably lessened, and individual existence is not so frequently abridged. The victims of rectal diseases are to be congratulated that this branch of science, or pseudo-science, has sufficiently advanced, that it now occupies the serious attention of the most progressive and intelligent men.

The Lister methods of that day have been so changed and improved that they now seem very crude. The value of thorough cleanliness, asepsis, and the antiseptic influence of certain drugs, is of immeasurable value. It is now understood that the recto-anal area can be placed in a surgically clean condition, and that there need be no fear following operative interference. In not a few instances, it obtains that relief is dependent on rectal surgery, when the subjects are unfit for narcosis produced from a general anesthetic, in cases of cardiac, pulmonic or nephritic disease, making it hazardous to use general anesthesia. Sometimes it would seem that this danger of the uses of an anesthetic is too lightly thought of, and consequently, the mortality rate is increased. Local anesthesia, under cocaine infiltration, for the most part, is satisfactory, and is a great convenience to the operator and a life-saving narcosis in many instances.

The palliative treatment of hemorrhoids by proctologists is largely a matter of enforcement, viz.: where they are not permitted the opportunity to relieve by radical methods. The operative methods of removing hemorrhoids are so well understood, simple and effective, that it is foolish to attempt to relieve them by drugs or palliative measures.

The Allingham, or ligature method, when correctly and carefully performed, is generally applicable, but is not so free from pain and so quickly convalesced from as the clamp and cautery method. Many regard the last mentioned method as the one to be preferred. I believe, however, that the enucleation method approaches nearest to the ideal in results, and that the retention of the plug is not so painful as some would have us believe.

Proctoscopic examination is of importance, and is a distinct advance in rectal work. It is of great assistance in determining disease beyond discovery by ordinary methods. It is of distinct service in diagnosis, and of great value in aiding treatment in not a few conditions.

There is more hope for the ultimate cure of tubercular conditions; our better understanding of what environment means to these people will go far toward helping them to recovery, and there is not so much reason for a delayed recognition of the condition, which is of paramount importance.

I believe there is possibly a better understanding of syphilitic conditions, ulcerations, infiltrations and strictures, but the eternal dependence on anti-syphilitic treatment to resolve hyperplastic tissue is not so conspicuous, and progressive workers in this field realise that incision and excision are often necessary.

Concerning malignant and benign growths, the surgical rules that apply in other anatomical regions apply here. Early discovery and early removal is the only hope, as we all know, in malignant conditions, and as an advance, the removal of cancerous growths not within easy reach from below may be dealt with from above, or suprapubicly, and just here it may not be inopportune to remark that it is to be believed that ere long it will be realised by the average physician that the removal of the rectum per se, is not as disastrous a matter as it is sometimes made to appear, especially since it is known that muscular transplantation will preserve more or less perfectly the function of the sphincters. The development of the technic essential to produce sphincteric power, will relieve rectal extirpation of one of its most unpleasant features and render less hesitant many sufferers who should have the benefit of the operation.

Another matter of progressive interest is that colonic or rectal ptosis is amenable to intrapelvic or intraabdominal fixation, bringing relief that in some instances cannot be hoped for by any other method of interference.

After all, the most encouraging sign is that the profession recognises the fact that proctologists have a legitimate right to exist as specialists, and that diseases in the ano-rectal region deserves the same consideration as elsewhere. With the elimination of indifference, etheticism, modesty, the more universal belief in the necessity of early examination and diagnosis, we can but hope for greater progress and more relief to suffering humanity.

When I consider the personnel of this Association, I am quite confident of the perpetuity of proctology as a distinct entity and am equally sure the progression in this special field of work will be in keeping with that in other specialties.

A Review of Proctologic Literature from May, 1908, to May, 1909, by Samuel T. Earle, M.D., Baltimore.

Among the interesting conditions referred to in the review by the author, were the following: "Congenital Idiopathic Dilatation of the Colon" (Hirschprung's Disease). In Dr. Finley's report of his case he reviewed the literature of the subject to January 1, 1908, and collected some 206 cases, after which he stated that while to Hirschprung belongs the credit of having first called attention to this disease, a number of cases had been found in the literature antedating his classical description. In the article Dr. Finley discussed the various hypothesis as to the etiology of the disease and some ten theories, which have been suggested from time to time, as the causation of the malady, including that of hypernutrition, which was the author's principal theory. His conclusions as to the etiology of the disease were that no one theory apparently explained every case; that each one explains some.

The symptomatology was described and a complete clinical picture of the disease given with a list of a series of cases discussed in the Johns Hopkins Hospital—eleven in all. Regarding the treatment, the author concludes that no one plan seems applicable to all cases and suggests the method employed in his own case as perhaps the one most applicable to the large proportion of cases, to-wit: a preliminary enterostomy; than a colostomy some months subsequently; finally, a complete excision of the affected portion. This artificial anus is left open until after the success of the proceeding steps are assured when it should be closed under cocaine anesthesia.

Dr. Earle in his report alluded to another case of "Idiopathic Dilatation of the Rectum and Colon as far as the Hepatic Flexure," which was reported by H. Morely Fletcher and H. Betham Robinson, M.S. (Clinical Society's Transactions, Vol. XL., p. 80).

Another case of interest reported was that of a "Sarcoma of the Rectum in a Boy aged ten years," by Cecil Rountree (Proceedings Royal Society of Medicine, February, 1908). The pathological examination showed the tumor to be a mixed cell sarcoma. Of 596 cases analysed in the Cancer Research Laboratory, of the Middlesex Hospital Reports, there were only six cases under thirty years of age,—the age of the youngest, a boy of sixteen years,—who had a sarcoma of the rectum. There are likely to be many metastasis in sarcoma of the rectum. This malady is rare at any age.

Attention was called to the method of Dr. Dudley Roberts of Brooklyn, (*Medical Record,* Vol. 72, p. 985), for "Gradual Painless Dilatation of the Anal Canal by Dilatable Rubber Bags," which appealed to Dr. Earle forcibly as a very satisfactory means of accomplishing the purpose designed.

Attention was called to the article of Dr. Charles O. Files, of Portland, Maine, (*New York Medical Journal,* Vol. 87, p. 1154), in which he considers that there are two important factors that should be studied in connection with the "Treatment of Pruritus Ani." These are an analysis of the contents of the rectum and the physical condition and mechanical efficiency of the sphincter ani muscles—external and internal.

The normal feces contains about 73 per cent of water. This water holds in solution various volatile, fatty acids, and probably other irritating excrementitious substances. During the retention of the feces in the rectum a considerable portion of the water disappears. In prolonged constipation, the feces become hard and dry, some of the fluid passes by osmosis into the cellular tissue about the anus and thence to the skin. The liquid feces are very often irritating to the mucous membrane of the anus, and causes an intense burning sensation. When this acrid solution is absorbed into the cellular tissue, it causes an irritation of the skin, and we call that irritation, pruritus ani.

The sphincter muscle as long as it remains in a normal condition prevents the passage of any appreciable amount of fluid through it. When, however, the action of the sphincter is made somewhat irregular by the pressure of a hemorrhoidal condition some of the fluid leaks through the anus and causes pruritus by direct contact. The skin about the anus is often found to be moist in persons having hemorrhoids.

Dr. F. W. Dudley, of Manilla, P. I., (*Jour. of American Medical Association,* Vol. 51, p. 991), reports a "New Bloodless Method of Amputating the Anus and Rectum." A description of the same being given.

Dr. W. Ernest Miles, (*London Lancet,* 1908, Vol. 2, p. 1812), reviews the "Perineal Excision for Carcinoma of the Rectum and of the Pelvic Colon," and states that so far as he has been able to gather from the literature on the subject, the technic of previous operations seems to have failed in one important respect—namely, the complete eradication of the zone of upward spread of cancer from the rectum, whereby the chance of recurrence of the disease above the field of operation can be distinguished, if not entirely obviated. In his personal experience of fifty-seven such peritoneal operations, he found that recurrences took place in periods from six months to three years in fifty-four instances.

In order to ascertain the cause of his failures he made a postmortem examination of such of his patients who died and found that recurrence appeared in situations that were beyond the scope of removal from the peritoneum—namely, (*a*) the pelvic peritoneum; (*b*) the pelvic mesocolon; and (*c*) the lymph nodes situated over the bifurcation of the left common iliac artery. He considers that this area constitutes the zone of the upward spread of cancer of the rectum, the removal of which is just as imperative as is the thorough clearance of the axilla in cases of cancer of the breast, if freedom from recurrence is to be obtained.

The appreciation of this important fact, induced him two years ago, to abandon the perineal methods of excision of the rectum and to substitute, therefor an abdominal method, comparable to those methods of performing abdominal hysterectomy, known as the Wertheim and the Kronig-Wertheim. He then gives the technic of his operation in full, and has formulated what he considers certain essentials, which must be strictly adhered to, if satisfactory results are to be obtained—namely, (1) that an abdominal anus is a necessity; (2) that the whole of the pelvic colon, with the exception of the part from which the colostomy is made, must be removed because its blood supply is contained in the zone of the upward spread; (3) that the whole of the pelvic mescolon below the point where it crosses the common iliac artery, together with a strip of peritoneum, at least an inch wide on either side of it, must be cleared away; (4) that the group of lymph nodes situated over the bifurcation of the common iliac artery are in all instances to be removed; and, lastly (5) that the peritoneal portion of the operation should be carried out as widely as possible, so that the lateral and downward zones of spread may be effectively extirpated.

B. G. A. Moyinham, Leeds, Eng., (*Surgery, Gynecology and Obstetrics,* 1908, Vol. 6, p. 463), calls special attention to the "Frequent Recurrences After Removal of Carcinoma from the Upper Rectum and Sigmoid," and also for the necessity of inguinal colostomy on account of the sacrifice of a large portion of the bowel in perhaps a large majority of cases.

Treatment of Pruritus Ani, with a Consideration of its Pathology and Etiology, by William M. Beach, A.M., M.D., of Pittsburg.

The following conclusions were drawn by the writer:

1. That pruritus ani occurs in mild and severe forms; mostly in middle life; the mild type with simple pruritus, the severe type with marked eczema and skin changes.

2. Certain aberrations in general metabolism, or in adjacent structures are simply incidental and should be considered as complications.

3. Intrarectal growths, as hemorrhoids, adenomas, and the like, or the presence of parasites are contributory.

4. The distinct pathogenesis of pruritus ani consists of single or multiple burrowings from the anal pockets, emitting a serous or sero-purulent substance, which sinus may be complete or blind and is always accompanied by proctitis, and frequently by cryptitis, and small ulcers at the ano-rectal line.

5. These sinuses when complete are the sequelæ to an abscess history, but the origin of the blind recesses is in doubt, and yet it is not unlikely due to an infection by the colon bacillus.

6. The treatment is surgical for the purpose of obliterating the sinuses, correcting a rigid sphincter when necessary, and curing the proctitis and ulceration.

7. Gastrointestinal and general metabolic disturbances must be met by rational measures.

Pruritus Ani, its Etiology and Treatment, by T. Chittenden Hill, M.D., Boston.

I am convinced that pruritus ani was practically always caused by some local lesions of the pelvic colon or rectum, which produced an unnatural moisture about the anal region.

He said the most common sources of irritation, in the order of their frequency, were as follows: (1) superficial ulcerations and abrasions of the anal canal. This lesion he found in about 75 per cent. of all cases and attributed the frequency of its occurrence to the method of fusion of the proctodeum with the blind end of the bowel.; (2) rectitis and sigmoiditis, which are the sequellæ of habitual constipation, often bring about a pruritus, since the passage of flatus allows a small quantity of mucus to escape; (3) hypertrophied anal papillæ and inflammation of the crypts of Morgagni are more often the cause of pruritus ani than is generally admitted; (5) small polyps of the anal canal, protruding internal piles, prolapse of the rectum and anal fissure, do occasionally produce itching about the anus, but it is exceptional to find them the sole cause of chronic pruritus ani.

He stated that in order to attain permanent results, it was essential that the treatment be directed to the removal of the exciting causes. At the same time the skin in the immediate vicinity of the anus should receive appropriate treatment since it is nearly always in a state of acute inflammation from scratching or so much infiltrated and thickened as to require stimulating applications,—nitrate of silver and ointments, in order to bring about a return of a normal epidermis.

Ball's Operation in the Treatment of Cases of Pruritus Ani with Report of a Case in which Necrosis of the Flap Occurred, by Louis J. Krouse, M.D., of Cincinnati.

The case reported was that of a severe intractable case of pruritus ani in a man well advanced in years who underwent the above operation for pruritus with the result of having the anal flap necrose. He went into the pathology as to the cause of the necrosis and came to the conclusion that the trouble lay in the poor supply of blood to the anal flap. He claimed that there is no anastomosis between the bloodvessels from within the anus and those of the skin. The writer called attention to the fact that Sir Charles Ball's operation has recently been modified so as to prevent sloughing of the anal flap.

A new method of operating was proposed by the author which is somewhat different from that of Sir Charles Ball and of that of Dr. Thomas Charles Martin, and consists: first, in doing away with the elliptical incision which cuts off the greater part of the circulation from the diseased area; and, secondly, in making six to eight linear incisions through the skin into the substaneous connective tissue. These linear incisions, beginning at a point outside of the point of irritation, follow the course of the radii of a circle whose center is the anal canal. The skin lying between the adjacent radii are then undercut until the whole affected area is undermined. Should the dissection be difficult and more room be needed, every alternate flap could then be loosened at the anal margin and dissected outwards toward the periphery. After all the adhesions are loosened and the bleeding has been stopped, the parts are again replaced and sutured.

The advantages of this operation over the original one of Ball, lie mainly in the better nourishment of the flap. The blood must come from the circumference and must radiate towards the anal canal.

A Consideration of the Prophylaxis and Treatment of Cicatricial Rectal Stricture, by Alois B. Graham, A.M., M.D., Indianapolis.

Opinions were based upon the results obtained in the treatment of fifty-five cases. He stated that prophylaxis implies a careful rectal examination; a careful rectal examination implies an early diagnosis; an early diagnosis implies correct treatment, and correct treatment implies the prevention of a stricture.

When cicatricial rectal stricture is diagnosticated, surgical intervention is indicated. In cases where there is no danger of infection, excision should be the choice of all the surgical measures at our command. If successful, its results are ideal because of the fact that it effects a cure by the complete removal of the stricture. In cases where it is not safe to practise the excision method (and there are many such cases) complete posterior proctotomy or colostomy, either alone or combined, should be performed. While neither of these surgical measures have effected an authentic cure, yet they undoubtedly can and have effected a symptomatic cure. Gradual dilatation should be employed only in cases of small annular stricture. The excision methods needs no defense as its results are all that could be desired. As for the other surgical methods the writer was not at all pessimistic as to the results which can be obtained, if they are followed by correct and systematic after-treatment.

THE USE OF SPINAL ANESTHESIA IN RECTAL SURGERY, by Collier F. Martin, M.D., Philadelphia.

Who reported eighty-seven cases in which tropacocain and stovaine were employed. The technic was given in detail. The method is not recommended where the hips of the patient have to be elevated.

Of the 87 cases, 57 were either frankly tubercular or the condition was suspected, 16 were alcoholics, 4 had anemia with from 35 per cent. to 60 per cent. of hemaglobin, 2 had sepsis, 2 cachexia, 2 were suffering from general debility and old age, 3 had cardiac complications and 1 refused to take ether.

The conditions operated upon were as follows: abscess and fistulæ, 54; hemorrhoids, 21; rectal stricture, 2; sacral sinus, 1; fissure with fistula, 2; gangrenous cellulitis, 2; anal condylomata, 2; rectal carcinoma (perineal excision) 2, and Ball's operation for pruritus ani, 1.

The only complications observed were headache eighteen times, coming on from one to three days after operation. Only three cases had severe headache lasting over one or two days. A few cases complained of some stiffness of the back of the neck and shoulders. One patient developed a temporary oculo-motor palsy which recovered under treatment. In two cases spinal fluid was not obtained because of the difficulty in inserting the needle with spinal deformity present.

Spinal anesthesia was selected in cases with pulmonary tuberculosis to avoid the congestion following the use of ether. Alcoholics were also found easier to manage than when ether was used.

Under spinal anesthesia, the sphincters are completely relaxed, there is no muscular spasm and there is an entire absence of the venous engorgement and swelling of the tissues so often seen while the patient is under ether. Bleeding is not as profuse and is more easily controlled, since all parts of the rectal cavity are as accessible as their anatomy will permit. The complete muscular relaxation reduces the traumatism to the tissues.

Spinal anesthesia is at its best when used in operations about the rectum and genitourinary tract. Careful selection of cases, drugs of uniform strength and purity, and a careful technic will do much to reëstablish the confidence of the surgeon in this method of producing anesthesia.

VAGINAL ANUS IN THE ADULT, WITH REPORT OF TWO CASES, by Louis J. Hirschman, M.D., Detroit.

Dr. Hirschman reported two cases of imperforate anus with the anomalous opening occurring in the lower part of the vagina, both occurring in adults. He successfully operated in both cases, restoring the anal outlet to its normal position with a good functional result in both cases. His first case was aged 25, unmarried, and until a few months before examination did not know that she was anatomically different from other young women. She was brought up by a maiden aunt who, while realising that her charge was not normal, felt that as long as she was having regular bowel movements, she would put off any operative interference until later in life.

The operation in this case consisted in closing the vaginal anal orifice after dissecting the rectum free from the vaginal septum. There being present an infantile sphincter muscle at the normal anal site, an incision was made through the center of this, and by blunt dissection the tissues between it and the blind end of the rectum were separated. The rectum was then pulled down, opened and sutured to the integument. The perineum was not split open nor was the sphincter divided. A good functional result followed.

His second case was also unmarried, 23 years of age. The case was very similar to Case I., except that there was an overdevelopment of the sphincter vaginæ which gave her good fecal control. There was present in this case a small fistula connecting the anus and vulva but not communicating with the rectum. In this case the perineum was split and the fistula dissected out. The vaginal anus was dissected free and brought down to the normal anal site in a manner similar to that pursued in Case I. The perineum was then repaired as in an ordinary perineorrhaphy. The functional result in this case was also good. The author concludes from his experience with these two cases, and realising the very high mortality from operations for imperforate anus, in infants, that where there is some abnormal outlet for the feces present, it is far better to allow patients to go on in their abnormal condition until they grow old and strong enough for surgical interference and the correction of nature's failure.

Tubercular Fistula with Extensive Infiltration with Specimen Exhibited, by Samuel T. Earle, M.D., Baltimore.

Reported a case of tubercular ischio-rectal fistula, which on the skin surface resembled an acute inflammatory condition ready to break down, yet when opened, it proved to be a dense mass of fibrous tissue with only a few tracts of necrotic tissue running through it.

The patient was a policeman, age 45; robust and of a ruddy color, weighing 180 pounds; no cough, no history of pulmonary trouble. Patient admited to hospital December 29, 1906.

The left buttock was very much swollen and inflamed; there were several fistulous openings on its surface, which could not be followed far beneath the skin, and there was one of them that opened just to the right of the anterior commissure, into the anal canal. Upon laying open the buttock between two of the openings, there was exposed a mass of white fibrous tissue that seemed to be encapsulated,—except at points which apparently were necrotic,—which was adherent to the subcutaneous tissue. Supposing it to be a tumor, which had broken down in places, an incision was made, on either side near each lateral border, for the purpose of removing it, which was done. The mass measured 6x3x2 inches.

It ran down to and some went between the muscles of the buttock, and in one or two instances involved the same. The tract from the inner margin of the mass, to the opening in the anal canal, was then laid open and packed with gauze. The cavity left was so large that sutures were introduced to draw the edges partially together, and to hold in the packing. These were supplemented by adhesive strips.

After the mass was removed, it was found to be composed principally of fat, with here and there a sinus which was surrounded by dense fibrous tissue from one-quarter to one-half inch thick, and there were found several large larva, supposedly of flies, deep down in the sinuses of the growth. The tapering, tail-like process, that extended over the trochanter major, was composed principally of muscle.

Upon microscopical examination, the growth proved to be tubercular. The patient made a slow but complete recovery. The large cavity filled in completely. The patient is now perfectly well and robust.

Fistula in the Posterior Anal Commissure, by J. Coles Brick, M.D., Philadelphia.

Who stated that the anatomy of the posterior anal commissure is of such peculiar arrangement that ulcers or fistulas, in this region frequently do not granulate in a proper manner.

The greater part of the external sphincter muscle arises from the coccyx, and after forming the ano- coccygeal body of Symington, passes around the anus, forming a Y-shaped or triangular cul-de-sac at the posterior anal commissure, making this the weakest part of the anal circumference. The levator ani muscle is separated from the coccygeus muscle by a cellular interspace, rendering possible an easy extension of pyogenic organisms.

In ulcerations or small fistulas in the posterior anal commissure, it is the writer's custom to make a triangular incision with the apex toward the anus, rather than an antero-posterior cut. In cases of fissure in this commissure, two incisions, 1/8 of an inch deep are made down into the sphincter muscle on each side of the fissure, all fibrous tissue being removed from the fissure itself.

The physiological action is, that during defecation, the lateral fibers of the sphincter forming the triangular space are at rest, due to their division; thus saving distension of this space, and consequently no interference with healing.

Modified Technic in Resection of the Rectum, by J. Rawson-Pennington, M.D., Chicago.

Numerous illustrations were shown by the author, intended to serve as demonstrations designed and employed by himself and Dr. Gronnerud in resection of the rectum in a special case. The growth for which the method was employed extended upward from the upward border of the levator and muscle for about two and one-half inches.

Aperineoraphy was first done, splitting the recto-vaginal septum back to Douglas cul-de-sac. The rectum as then dissected from its lateral and posterior connections upward until it could be pulled downward far enough to effect an end-to-end anastomosis, when the section, including the growth was removed.

The incision was closed with buried catgut-sutures, and silk-worm-gut for the skin. The posterior vaginal flap covering up, as it did, the operating field, prevents the urine, vaginal and uterine secretions, from coming in contact with the wound.

Abdominal Massage in the Treatment of Chronic Constipation, Etc., by T. L. Hazzard, M.D., B.S., Pittsburg.

The writer referred to the fact that general massage had been practised from very ancient times until the present for the relief of fatigue and for the purpose of increasing the flow of fluids in the bloodvessels, the lymph spaces and juice canals, by which more perfect elimination of waste is obtained and better assimilation brought about. Two conditions which, in his opinion the relief of will do away with two-thirds of the slight ailments as well as of some of the more serious ones. He began massage for the relief of chronic constipation and was much surprised to find the far reaching, adventitious effects produced. Among others, for example, that the chalky deposit in the joints in articular rheumatism, under careful, patient, persistent manual therapeutics as applied to the bowels, will entirely disappear more often than not.

Mentioned no particular method, saying that any good text-book would give the technic sufficiently well. This manipulation is recommended not only for chronic constipation, but also for the relief of coprostasis for which operation it is very frequently done.

After indicating more of the benefits and some of the dangers of the method, the writer said that if this treatment called for more time than the physician or surgeon could spare, it had better be left off altogether, although the patient would surely lose a very great benefit. The paper closed with the remark that doubters as to the very great advantages which will accrue to the sick, in many, many ailments, has but to practise careful and intelligent massage to be convinced.

